# Absence of influence of gender and *BMPR2 *mutation type on clinical phenotypes of pulmonary arterial hypertension

**DOI:** 10.1186/1465-9921-11-73

**Published:** 2010-06-10

**Authors:** Barbara Girerd, David Montani, Mélanie Eyries, Azzedine Yaici, Benjamin Sztrymf, Florence Coulet, Olivier Sitbon, Gérald Simonneau, Florent Soubrier, Marc Humbert

**Affiliations:** 1 Faculté de Médecine, Université Paris-Sud, Kremlin-Bicêtre, F-94276, France; 2 Service de Pneumologie et Réanimation Respiratoire, Centre National de Référence de l'Hypertension Pulmonaire Sévère, Hôpital Antoine Béclère, Assistance Publique-Hôpitaux de Paris, Clamart, F-92140, France; 3 INSERM U999, Hypertension Artérielle Pulmonaire: Physiopathologie et Innovation Thérapeutique, Centre Chirurgical Marie-Lannelongue, Le Plessis-Robinson, F-92350, France; 4 Laboratoire d'Oncogénétique et Angiogénétique Moléculaire, UMRS 956 INSERM, Université Pierre et Marie Curie-Paris 6, Groupe Hospitalier Pitié-Salpétrière, Paris, F-75651, France

## Abstract

**Background:**

Previous studies indicate that patients with pulmonary arterial hypertension (PAH) carrying a mutation in the bone morphogenetic protein receptor type 2 (*BMPR2*) gene, develop the disease 10 years earlier than non-carriers, and have a more severe hemodynamic compromise at diagnosis. A recent report has suggested that this may only be the case for females and that patients with missense mutations in *BMPR2 *gene have more severe disease than patients with truncating mutations.

**Methods:**

We reviewed data from all patients with PAH considered as idiopathic and patients with a family history of PAH, who underwent genetic counselling in the French PAH network between January, 1^st ^2004 and April, 1^st ^2010. We compared clinical, functional, and hemodynamic characteristics between carriers and non-carriers of a *BMPR2 *mutation, according to gender or *BMPR2 *mutation type.

**Results:**

PAH patients carrying a *BMPR2 *mutation (n = 115) were significantly younger at diagnosis than non-carriers (n = 267) (35.8 ± 15.4 and 47.5 ± 16.2 respectively, p < 0.0001). The presence of a *BMPR2 *mutation was associated with a younger age at diagnosis in females (36.4 ± 14.9 in *BMPR2 *mutation carriers and 47.4 ± 15.8 in non-carriers, p < 0.0001), and males (34.6 ± 16.8 in *BMPR2 *mutation carriers and 47.8 ± 17.1 in non-carriers, p < 0.0001). *BMPR2 *mutation carriers had a more severe hemodynamic compromise at diagnosis, but this was not influenced by gender. No differences in survival and time to death or lung transplantation were found in male and female PAH patients carrying a *BMPR2 *mutation. No differences were observed in clinical outcomes according to the type of *BMPR2 *mutations (missense, truncating, large rearrangement or splice defect).

**Conclusion:**

When compared to non-carriers, *BMPR2 *mutation carriers from the French PAH network are younger at diagnosis and present with a more severe hemodynamic compromise, irrespective of gender. Moreover, *BMPR2 *mutation type had no influence on clinical phenotypes in our patient population.

## Background

Pulmonary arterial hypertension (PAH) is a severe disease affecting small pulmonary arteries, with a progressive remodeling leading to elevated pulmonary vascular resistance and right ventricular failure [1-3]. PAH can be idiopathic, heritable or associated with drug or toxin exposure or other conditions such as connective tissue diseases, human immunodeficiency virus infection, congenital heart diseases, and portal hypertension [1,2]. Germline mutations in the bone morphogenetic protein receptor type 2 (*BMPR2*) gene are detected in 10 to 40% of idiopathic PAH and in 58% to 74% of patients with a family history of PAH [4-6]. Accumulated evidence indicates that PAH patients carrying a *BMPR2 *mutation develop PAH approximately 10 years earlier than non-carriers, with a more severe hemodynamic compromise at diagnosis, and are less likely to respond to acute vasodilator testing [4-6]. In addition, few cases of PAH in *ACVRL1 *and *Endoglin *mutants have been reported [6-9]. PAH patients with an *ACVRL1 *mutation are characterized by a younger age at diagnosis and death, as compared to PAH patients without *BMPR2 *and *ACVRL1 *mutation [6].

A recent report in *Respiratory Research *by Austin and colleagues [10] showed that PAH patients with missense mutations in *BMPR2 *gene had more severe disease than patients with truncating mutation with a significant younger age at diagnosis, a younger age at death and a shorter survival from diagnosis to death or lung transplantation. Moreover, they demonstrated a statistically significant difference in age at diagnosis between carriers and non-carriers, and subgroup analysis revealed this to be the case only for females [10].

To test the influence of gender in patients from the French PAH network, we decided to compare clinical, functional, and hemodynamic characteristics between PAH patients carriers of a *BMPR2 *mutation and patients without identified mutation, according to gender. To analyse the influence of the type of *BMPR2 *mutation in clinical outcomes of PAH, clinical phenotypes were compared between *BMPR2 *missense mutation carriers, *BMPR2 *truncating mutation carriers and patients carriers of a large rearrangement or a splice defect in the *BMPR2 *gene.

## Methods

### Patients

We reviewed data from all patients with PAH considered to be idiopathic and patients with a family history of PAH, who were tested for *BMPR2 *and *ACVRL1 *mutations, seen in the French PAH network (Université Paris-Sud 11, Hôpital Antoine-Béclère, Clamart, France) between January, 1^st ^2004 and April, 1^st ^2010. In accordance with the guidelines of the American College of Chest Physicians [11], patients tested for *BMPR2 *or *ACVRL1 *mutations signed written informed consent and underwent genetic counselling. A diagnosis of PAH was defined by hemodynamic measurement during right-heart catheterization (see below). Idiopathic PAH was recognized after ruling out all causes of pulmonary hypertension, summarized in the revised classification, and by determining no other PAH cases in the family [4]. Familial PAH was recognised if there was more than one confirmed case in the family. PAH patients with an *ACVRL1 *mutation (n = 9), a suspected or confirmed pulmonary veno-occlusive disease (n = 55), or a family history of PAH without identification of either *BMPR2 *or *ACVRL1 *mutation (n = 13) were excluded in order to limit the risk of misclassification. All clinical characteristics at PAH diagnosis and follow-up were stored in the Registry of the French PAH Network [12]. This Registry was set up in agreement with French bioethics laws (French Commission Nationale de l'Informatique et des libertés), and patients gave their consent to be included [4,12].

### Hemodynamic measurements and 6-minute walk distance

PAH was defined as a mean pulmonary arterial pressure (mPAP) ≥25 mmHg associated with a normal pulmonary capillary wedge pressure (PCWP). Hemodynamic evaluation by right heart catheterization was performed at baseline in all subjects according to our previously described protocol [13,14]. Patients with left heart diseases and passive increase of mPAP secondary to increase cardiac output were excluded from the study. The mPAP, PCWP, right atrial pressure (RAP) and mixed venous oxygen saturation (SvO_2_) were recorded during right-heart catheterization. Cardiac output (CO) was measured by the standard thermodilution technique. The cardiac index (CI) was calculated as the CO divided by the body surface area and systolic index as the CI divided by cardiac frequency. Indexed total pulmonary resistance (TPRi) and indexed pulmonary vascular resistance (PVRi) were calculated as (mPAP)/CI and (mPAP-PCWP)/CI respectively, and results were expressed as mmHg/L/min/m². Baseline hemodynamic data and response to short-term vasodilator nitric oxide (NO) obtained through right heart catheterization were performed for all subjects. A NO challenge (10 ppm for 5-10 minutes) was used and a positive acute response was defined as a decrease in mPAP of more than 10 mmHg compared to the baseline mPAP to reach a mPAP lower than 40 mmHg associated with a normal or increased CO, as previously described [1,2,13]. A non-encouraged 6-minute walk test according to the American Thoracic Society recommendations was performed [15].

### Screening of point mutations and large rearrangements of *ACVRL1 *and *BMPR2 *genes

Human genomic DNA was prepared from whole blood samples. Amplification of the entire coding sequence and intronic junctions of the *ACVRL1 *and *BMPR2 *genes was performed on 50 ng of genomic DNA from each individual. Genetic variation of *BMPR2 *and *ACVRL1 *sequence and rearrangements of one or more exons of the *ACVRL1 *and *BMPR2 *genes was detected as previously described [4,6].

### Statistical analysis

We compared demographic and clinical features, age at diagnosis of PAH, according to gender and *BMPR2 *mutation status, or according to type of *BMPR2 *mutation with the use of Chi-2, ANOVA post Hoc test and Mann Whitney as appropriate. Survival and time to death or lung transplantation were analysed with the use of Kaplan Meier Analysis. A p value of less than 0.05 was considered to indicate statistical significance.

## Results

### Influence of gender on phenotypes

**Figure 1:**
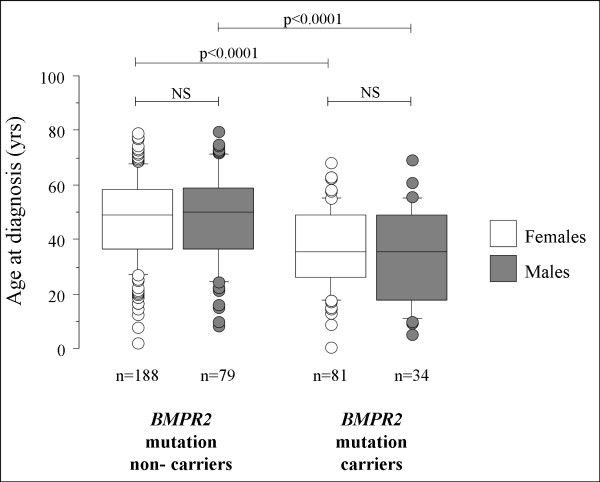
**Age at PAH diagnosis in females and males according to *BMPR2 *mutation status**. *BMPR2*: bone morphogenetic protein receptor type 2, PAH: pulmonary arterial hypertension.

**Figure 2:**
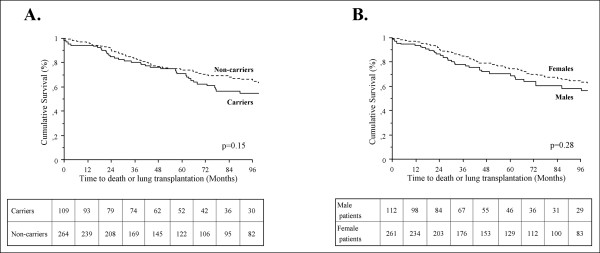
**Time to death or lung transplantation in all patients according to *BMPR2 *mutation status (A) or gender (B)**. *BMPR2*: bone morphogenetic protein receptor type 2.

**Figure 3:**
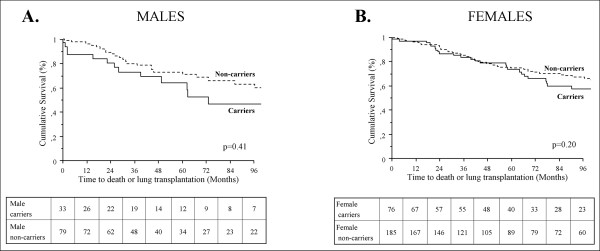
**Time to death or lung transplantation in males (A) and in females (B) according to *BMPR2 *mutation status**. *BMPR2*: bone morphogenetic protein receptor type 2.

In the French PAH network, 382 PAH patients with idiopathic PAH or with a family history of PAH, corresponding to 113 males and 269 females, were screened for *BMPR2 *mutations. Interestingly, the same proportion of *BMPR2 *mutation carriers was observed in males (n = 33, 30.1%) and females (n = 81, 30.1%). As previously reported [4,6,10], PAH patients carrying a *BMPR2 *mutation were significantly younger at diagnosis than non-carriers (35.8 ± 15.4 and 47.5 ± 16.2, p < 0.0001). This was the case in females (36.4 ± 14.9 in *BMPR2 *mutation carriers and 47.4 ± 15.8 in non-carriers, p < 0.0001) (Figure 1), as well as in males (34.6 ± 16.8 in *BMPR2 *mutation carriers and 47.8 ± 17.1 in non-carriers, p < 0.0001) (Figure 1). As previously reported [4,6], *BMPR2 *mutation carriers had a more severe hemodynamic compromise at diagnosis, but this was not influenced by gender (Table 1). Although *BMPR2 *mutation carriers are younger at diagnosis than non-carriers, survival and time to death or lung transplantation is broadly similar between carriers and non-carriers, suggesting a similar evolution of established disease in heritable and idiopathic PAH (Figure 2A). When comparing survival and time to death or lung transplantation in *BMPR2 *mutation carriers and non-carriers according to gender, no statistically significant differences were observed between males and females (Figure 3A and 3B). Although males and females have the same age at diagnosis (43.8 ± 18.0 and 44.2 ± 16.3 respectively, p = 0.86), and have also the same age at death (50.4 ± 22.9 and 49.0 ± 16.9 respectively, p = 0.74), we observed a trend for more severe prognosis of the disease in males with a shorter time to death or lung transplantation (Figure 2B), and particularly in males carrying a *BMPR2 *mutation (Figure 3A).

**Table 1:** Baseline hemodynamic characteristics of male and female patients carrying a *BMPR2 *mutation.

	Male patients			Female patients		
	***BMPR2 *mutation non-carriers** **n = 79**	***BMPR2 *mutation carriers** **n = 34**	**p**	***BMPR2 *mutation non-carriers** **n = 188**	***BMPR2 *mutation carriers** **n = 81**	**p**

**Age at diagnosis, *yrs *(mean ± SD)**	47.8 ± 17.1	34.6 ± 16.8	< 0.0001	47.4 ± 15.8	36.4 ± 14.9	< 0.0001
**NYHA**						
**I-II**	21 (26.6%)	6 (17.6%)	< 0.01	27 (14.4%)	11 (13.6%)	0.90
**III**	57 (72.2%)	21 (61.8%)		135 (71.8%)	55 (67.9%)	
**IV**	1 (1.3%)	6 (17.6%)		20 (10.6%)	10 (12.3%)	
**Six-minute walk test distance, *m***	394 ± 96	377 ± 98	0.49	328 ± 119	343 ± 102	0.35
**mPAP, *mmHg***	57 ± 14	63 ± 13	0.03	56 ± 14	62 ± 13	0.0007
**RAP, *mmHg***	8 ± 5	8 ± 6	0.81	8 ± 5	8 ± 5	0.70
**PCWP, *mmHg***	9 ± 3	8 ± 3	0.39	8 ± 3	8 ± 3	0.84
**CI, *L/min/m²***	2.71 ± 0.83	2.17 ± 0.60	0.0003	2.45 ± 0.66	2.07 ± 0.62	< 0.0001
**PVRi, *mmHg/L/min/m²***	19.6 ± 9.3	21.9 ± 13.5	0.29	20.5 ± 8.5	25.1 ± 11.1	0.0015
**SvO_2, _*%***	67 ± 8	59 ± 9	0.0015	61 ± 10	59 ± 10	0.09
**Acute vasodilator responders, *%***	9 (11.4%)	1 (2.9%)	0.20	27 (14.4%)	1 (1.2%)	< 0.01

*BMPR2*: bone morphogenetic protein receptor type 2, PAH: pulmonary arterial hypertension, NYHA = New York Heart Association, mPAP = mean pulmonary artery pressure, RAP = right atrial pressure, PCWP = pulmonary capillary wedge pressure, CI = cardiac index, PVRi = indexed pulmonary vascular resistance, SvO2 = mixed venous oxygen saturation, NO = nitric oxide.

Results are expressed as mean ± SD

### Influence of *BMPR2* mutation type on phenotypes

**Figure 4:**
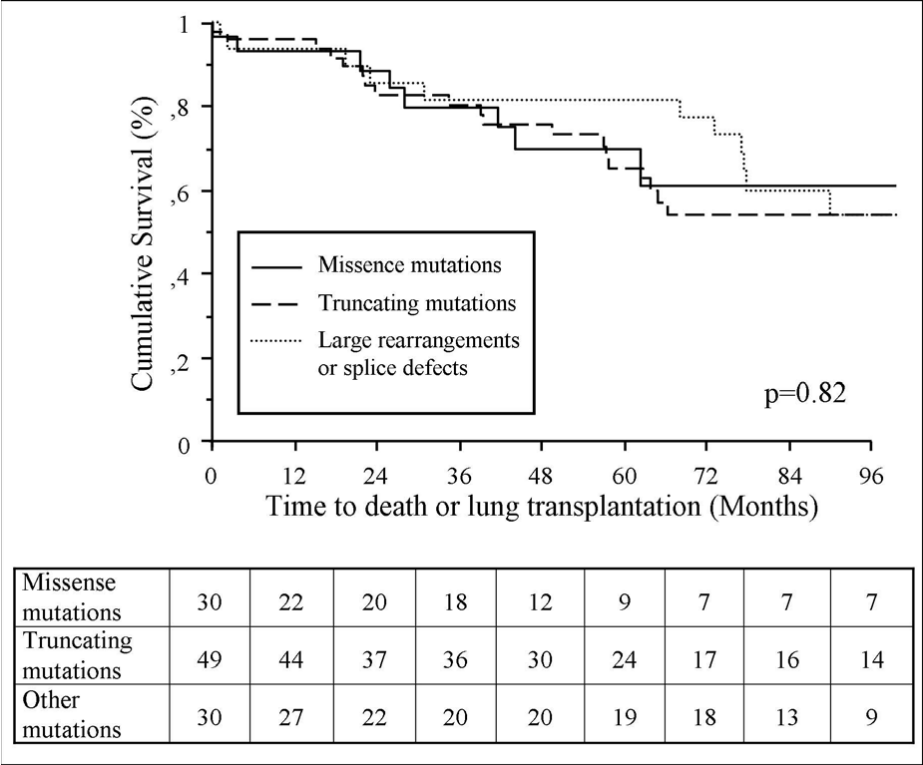
**Influence of *BMPR2 *mutation types on clinical outcomes of PAH patients**. Time to death or lung transplantation in *BMPR2 *missense mutation carriers, *BMPR2 *truncating mutation carriers and patients carriers of a large rearrangement or a splice defect. *BMPR2*: bone morphogenetic protein receptor type 2.

At the time of analysis, 115 patients were carriers of a *BMPR2 *mutation. Thirty two (27.8%) had a *BMPR2 *missense mutation, 51 (44.3%) a truncating *BMPR2 *mutation and 32 (27.8%) other *BMPR2 *mutations (12 splice defects and 20 large rearrangements) (Additional file 1). No differences in age at diagnosis were observed between *BMPR2 *missense mutation carriers, *BMPR2 *truncating mutation carriers and patients carrying a large rearrangement or a splice defect in the *BMPR2 *gene (35.8 ± 16.9, 37.3 ± 14.0 and 33.5 ± 16.3 respectively) (Table 2). Clinical, functional, and hemodynamic characteristics showed only a lower RAP and a higher CI in *BMPR2 *missense mutation carriers, when compared to *BMPR2 *truncating mutation carriers (Table 2). No statistically significant differences were observed in survival and time to death or lung transplantation between these 3 subgroups of *BMPR2 *mutation carriers (Figure 4).

**Table 2:** Baseline hemodynamic characteristics of *BMPR2 *mutation carriers according to mutation types.

	*BMPR2 *missense mutation carriers n = 32	*BMPR2 *truncating mutation carriers n = 51	*BMPR2 *large rearrangement or splice defect mutation carriers n = 32
**Age at diagnosis, *yrs *(mean ± SD)**	35.8 ± 16.9	37.3 ± 14.0	33.5 ± 16.3
**NYHA**			
**I-II**	5 (15.6%)	5 (9.8%)	7 (21.8%)
**III**	21 (65.6%)	37 (72.5%)	18 (56.3%)
**IV**	3 (9.4%)	7 (13.7%)	6 (18.8%)
**Six-minute walk test distance, *m***	382 ± 107	351 ± 91	325 ± 109
**mPAP, *mmHg***	63 ± 14	62 ± 12	62 ± 13
**RAP, *mmHg***	6 ± 5*	9 ± 5	8 ± 5
**PCWP, *mmHg***	8 ± 3	7 ± 2	8 ± 4
**CI, *L/min/m²***	2.31 ± 0.68^¶^	1.94 ± 0.41	2.16 ± 0.75
**PVRi, *mmHg/L/min/m²***	24.9 ± 10.8	25.0 ± 10.5	22.5 ± 14.9
**SvO_2, _*%***	60 ± 9	57 ± 10	60 ± 9
**Acute vasodilator responders, *%***	1(3.1%)	1 (1.9%)	0.9

*p = 0.02 compared to *BMPR2 *truncating mutation carriers

^¶^p = 0.01 compared to *BMPR2 *truncating mutation carriers

*BMPR2: bone morphogenetic protein receptor type 2, PAH: pulmonary arterial hypertension, NYHA = New York Heart Association, mPAP = mean pulmonary artery pressure, RAP = right atrial pressure, PCWP = pulmonary capillary wedge pressure, CI = cardiac index, PVRi = indexed pulmonary vascular resistance, SvO2 = mixed venous oxygen saturation, NO = nitric oxide*.

Results are expressed as mean ± SD

## Discussion

In this study, we show that *BMPR2 *mutation carriers are younger at diagnosis with a more severe hemodynamic compromise, but this is not influenced by gender. However, we observed a trend for more severe prognosis of the disease in males, and particularly in male patients carrying a *BMPR2 *mutation. This observation is consistent with our recent observation that PAH mortality is most closely associated with male gender [16]. Moreover, we compared *BMPR2 *missense mutation carriers, *BMPR2 *truncating mutation carriers and patients carrying a splice defect or a large rearrangement in the *BMPR2 *gene. We found no influence of mutation type on clinical phenotypes in *BMPR2 *mutation carriers with PAH.

Although no significant impact of gender was observed on age at diagnosis and outcomes in our patient population, it should be emphasized that PAH mostly occur in females, irrespective of *BMPR2 *mutation status (sex ratio females/males: 2.4 in *BMPR2 *mutation non-carriers, and 2.4 in *BMPR2 *mutation carriers). To explain overrepresentation of female patients it has been suggested that estrogens and estrogen metabolism might participate in the pathogenesis of PAH [17,18]. Estrogen is a potent mitogen of pulmonary vascular smooth muscle cells, and cytochrome P450 1B1 (CYP1B1), highly expressed in lung and particularly in endothelial cells, catalyzes the oxidation of estrogens to 2-hydroxy (2-OHE) and 4-hydroxy (4-OHE) estrogens. West et al. [17] have showed significantly decreased transcript levels of CYP1B1 in affected female compared to unaffected females with a *BMPR2 *mutation. They hypothesized that low level of CYP1B1 might result in increased local concentrations of estrogens, increasing the risk of PAH. Moreover, in extrahepatic tissue, oxidation of estrogens also occurs by hydroxylation at the C-16 position by other P450 enzymes, predominantly resulting in 16α-hydroxyestrone (16α-OHE1) which stimulates cellular proliferation by constitutively activating the estrogen receptor. Austin et al. [18] demonstrated a decrease of the 2-OHE/16α-OHE1 ratio in affected female compared to non-affected females with a *BMPR2 *mutation. Thus, altered estrogen metabolism could contribute to the penetrance of PAH in women and *CYP1B1 *could be a sex specific modifier gene.

Secondly, we compared clinical phenotypes of *BMPR2 *missense mutation carriers, *BMPR2 *truncating mutation carriers and patients carrying a splice defect or a large rearrangement in *BMPR2 *gene. Whereas Austin and colleagues [10] showed that PAH patients with missense mutations in *BMPR2 *gene had more severe disease than patients with truncating mutation with a significant younger age at diagnosis and a shorter survival from diagnosis to death or lung transplantation, we did not find any influence of mutation type on disease pattern or natural history among patients with *BMPR2 *mutation in the French PAH network. Austin and colleagues [10] hypothesized that this difference may be due to the fact that truncating mutations are degraded by nonsense-mediated decay (NMD), a mRNA surveillance mechanism that detects and degrades mRNA transcripts containing premature termination codons (PTCs), leaving only the wild-type mRNA detectable. By contrast, missense mutations escape NMD resulting in stable transcript that may produce dominant negative proteins [19,20]. Because some truncating mutations escape NMD, Austin et al. confirmed the activity of NMD pathway in *BMPR2 *truncating mutation carriers. *BMPR2 *truncating mutations escaping NMD pathway may represent a minority of cases, with only 7 out of the 62 *BMPR2 *truncating mutation carriers escaping NMD in this study [10]. Indeed, PTCs located more than 55 nucleotides upstream the last exon escape NMD. Analysis of all the truncating mutations in the French PAH network showed that 9 out of the 51 *BMPR2 *truncating mutation carriers had mutations predicted to escape NMD (Additional file 1). Nevertheless, if we excluded these 9 patients from the analysis of impact of *BMPR2 *mutation type on clinical outcomes, no significant differences were seen between *BMPR2 *missense mutation carriers, *BMPR2 *truncating mutation carriers and patients carrying a splice defect or a large rearrangement in *BMPR2 *gene. However, this analysis cannot include all *BMPR2 *mutations that could potentially escape NMD, because it has been demonstrated that truncating mutation may also escape NMD pathway by reinitiation of translation by use of an alternative translation start site downstream the PTC, producing a BMPRII protein of a lower molecular weight [19,20]. Indeed, other factors may influence the relation between *BMPR2 *mutation type and phenotype of PAH. It has been demonstrated that the level of expression of the wild-type *BMPR2 *allele may influence the clinical development of PAH in carriers of truncating *BMPR2 *mutations [21]. Moreover, different missense *BMPR2 *mutations could lead to different level of BMPRII protein expression on cell surface, depending on the level of cytosolic retention of BMPR-II transcript [22,23]. All these factors may explain the difficulty to correlate severity and penetrance of PAH to the *BMPR2 *mutation type.

The differences observed between our data and those reported by Austin et al. related to gender, may be explained by a selection bias with a low number of PAH patients without a *BMPR2 *mutation in the cohort published by Austin et al. (only 10 male *BMPR2 *mutation non-carriers as compared to 79 in the French PAH Network). This low number of male patients not carrying a *BMPR2 *mutation may be due to a preferential recruitment of patients with a family history of PAH in the study by Austin. Indeed, 74% of their patients had such a family history, as compared to 22% in the cohort of New York Presbyterian Pulmonary Hypertension Center [5] and 20% in the French PAH network [6]. This resulted to 72% of PAH patients carrying a *BMPR2 *mutation in the cohort reported by Austin and colleagues, as compared to 17% in the cohort from the New York Presbyterian Pulmonary Hypertension Center [5], and 25% in the French PAH network [6]. The lower number of *BMPR2 *mutation carriers in the cohort from the New York Presbyterian Pulmonary Hypertension Center [5] could be explained by the fact that patients with a considered idiopathic PAH were not screened for large rearrangement of *BMPR2*. In the French PAH network, all patients with idiopathic or familial PAH were screened for point mutations and large rearrangements of *BMPR2 *gene, to avoid selection bias.

The familial clustering in the French PAH network may explain the differences observed between our data and those reported by Austin et al. Indeed, familial clustering could impact data in terms of genes and environmental interactions that may alter disease expression beyond that of a single gene mutation. In contrast, in the French PAH network, 81 out of the 115 *BMPR2 *mutation carriers (70.5%) were the only cases reported in their families, while 14 families had 2 cases reported and 2 families had 3 cases reported. Thus the majority of PAH cases we studied were the only reported cases from their families and familial clustering represented only a minority of reported cases.

Finally, PAH patients with a family history of PAH without identification of either *BMPR2 *or *ACVRL1 *mutation (n = 13) were excluded from the analysis in order to limit the risk of misclassification, although their clinical and hemodynamic characteristics were broadly similar to those of *BMPR2 *mutation carriers (age at PAH diagnosis 38.9 ± 12.5 yrs, mPAP 64 ± 13 mmHg, PVRi 25.9 ± 9.5 mmHg/L/min/m²). Moreover, *ACVRL1 *and *Endoglin *are two other genes well known to predispose to PAH. However, similar approach was not possible in *ACVRL1 *and *Endoglin *mutation carriers because the number of patients was too low (1 male and 8 female *ACVRL1 *mutation carriers, and only one patient with an *Endoglin *mutation).

In conclusion, our data indicate that mutation type has no influence on disease pattern or natural history among patients with *BMPR2 *mutations. However *BMPR2 *mutation carriers (males or females) are younger at diagnosis with a similar evolution of the disease than non-carriers, leading to a younger age at death of *BMPR2 *mutation carriers compared to non-carriers.

## Abbreviations

2-OHE: 2-hydroxyestrogens; 4-OHE: 4-hydroxyestrogens; 16α-OHE1: 16α-hydroxyestrone; *BMPR2*: Bone morphogenetic protein receptor type 2; CYP1B1: Cytochrome P450 1B1; NMD: nonsense-mediated decay; PAH: Pulmonary arterial hypertension; PTC: Premature termination codons.

## Competing interests

The authors declare that they have no competing interests.

## Authors' contributions

BG and DM performed the statistical analysis and wrote the manuscript. ME, AY, BS, FC, GS, FS and MH helped to conception, design and wrote the manuscript. All authors read and approved the final manuscript.

## Supplementary Material

Additional file 1 **Details of *BMPR2 *Mutations**. Click here for file
