# Supplementary material for: Absence of influence of gender and BMPR2 mutation type on clinical phenotypes of pulmonary arterial hypertension
Source: Respir Res. 2010 Jun 10;11(1):73. doi: 10.1186/1465-9921-11-73 (PMC2898773; doi:10.1186/1465-9921-11-73)
Supplement: Additional file 1 — Details of BMPR2 Mutations. [file 1465-9921-11-73-S1.DOC]

**TABLE 1. DETAILS OF *BMPR2*** MUTATIONS.

| **Mutation**  **location** | **Nucleotide**  **change** | **Amino-acid**  **change** | **Mutation**  **category** |
| --- | --- | --- | --- |
| Exon 2 | c.197G>A | p.Cys66Tyr | Missense mutation |
| Exon 2 | c.197G>A | p.Cys66Tyr | Missense mutation |
| Exon 2 | c. 200A>G | p.Tyr67Cys | Missense mutation |
| Exon 2 | c.200A>G | p.Tyr67Cys | Missense mutation |
| Exon 2 | c.247G>A | p.Gly83Arg | Missense mutation |
| Exon 3 | c.280T>C | p.Cys94Arg | Missense mutation |
| Exon 3 | c.350G>C | p.Cys117Ser | Missense mutation |
| Exon 3 | c.367T>C | p.Cys123Arg | Missense mutation |
| Exon 3 | c.370A>G | p.Asn124Asp | Missense mutation |
| Exon 3 | c.377A>G | p.Asn126Ser | Missense mutation |
| Exon 3 | c.377A>G | p.Asn126Ser | Missense mutation |
| Exon 6 | c.830T>C | p.Leu277Pro | Missense mutation |
| Exon 7 | c.901T>C | p.Ser301Pro | Missense mutation |
| Exon 7 | c.901T>C | p.Ser301Pro | Missense mutation |
| Exon 7 | c.901T>C | p.Ser301Pro | Missense mutation |
| Exon 8 | c.1019T>C | p.Leu340Pro | Missense mutation |
| Exon 9 | c.1171G>A | p.Ala391Thr | Missense mutation |
| Exon 11 | c.1447T>C | p.Cys483Arg | Missense mutation |
| Exon 11 | c.1471C>T | p.Arg491Trp | Missense mutation |
| Exon 11 | c.1471C>T | p.Arg491Trp | Missense mutation |
| Exon 11 | c.1471C>T | p.Arg491Trp | Missense mutation |
| Exon 11 | c.1471C>T | p.Arg491Trp | Missense mutation |
| Exon 11 | c.1471C>T | p.Arg491Trp | Missense mutation |
| Exon 11 | c.1471C>T | p.Arg491Trp | Missense mutation |
| Exon 11 | c.1471C>T | p.Arg491Trp | Missense mutation |
| Exon 11 | c.1471C>T | p.Arg491Trp | Missense mutation |
| Exon 11 | c.1471C>T | p.Arg491Trp | Missense mutation |
| Exon 11 | c.1472G>A | p.Arg491Gln | Missense mutation |
| Exon 11 | c.1472G>A | p.Arg491Gln | Missense mutation |
| Exon 11 | c.1472G>A | p.Arg491Gln | Missense mutation |
| Exon 12 | c.2618G>A | p.Arg873Gln | Missense mutation |
| Exon 12 | c.2618G>A | p.Arg873Gln | Missense mutation |
| Exon 1 | c.48G>A | p.Trp16X | Truncating mutation |
| Exon 1 | c.48G>A | p.Trp16X | Truncating mutation |
| Exon 3 | c.255G>A | p.Trp85X | Truncating mutation |
| Exon 3 | c.274C>T | p.Gln92X | Truncating mutation |
| Exon 3 | c.320C>G | p.Ser107X | Truncating mutation |
| Exon 3 | c.339C>A | p.Tyr113X | Truncating mutation |
| Exon 3 | c.371dup | p.Asn124lysfsX6 | Truncating mutation * |
| Exon 3 | c.407_408del | p.Thr136AsnfsX10 | Truncating mutation |
| Exon 3 | c.408_412delAACAC | p.Pro138GlnfsX7 | Truncating mutation |
| Exon 4 | c.439C>T | p.Arg147X | Truncating mutation |
| Exon 4 | c.439C>T | p.Arg147X | Truncating mutation |
| Exon 4 | c.439C>T | p.Arg147X | Truncating mutation |
| Exon 4 | c.439C>T | p.Arg147X | Truncating mutation |
| Exon 4 | c.449dup | p.Ile151AsnfsX30 | Truncating mutation |
| Exon 4 | c.528delA | p.Gly177GlufsX10 | Truncating mutation |
| Exon 4 | c.528delA | p.Gly177GlufsX10 | Truncating mutation |
| Exon 5 | c.551_573del | p.His184ArgfsX8 | Truncating mutation * |
| Exon 6 | c.612delA | p.Lys204AsnfsX5 | Truncating mutation |
| Exon 6 | c.631C>T | p.Arg211X | Truncating mutation |
| Exon 6 | c.631C>T | p.Arg211X | Truncating mutation |
| Exon 6 | c.631C>T | p.Arg211X | Truncating mutation |
| Exon 6 | c.631C>T | p.Arg211X | Truncating mutation |
| Exon 6 | c.631C>T | p.Arg211X | Truncating mutation |
| Exon 6 | c.631C>T | p.Arg211X | Truncating mutation |
| Exon 6 | c.689_690del | p.Lys230SerfsX25 | Truncating mutation |
| Exon 6 | c.775delC | p.Arg259AlafsX3 | Truncating mutation |
| Exon 6 | c.782_783del | p.Ile261SerfsX4 | Truncating mutation |
| Exon 7 | c.928A>T | p.Arg310X | Truncating mutation * |
| Exon 7 | c.961C>T | p.Arg321X | Truncating mutation * |
| Exon 7 | c.961C>T | p.Arg321X | Truncating mutation * |
| Exon 7 | c.961C>T | p.Arg321X | Truncating mutation * |
| Exon 7 | c.961C>T | p.Arg321X | Truncating mutation * |
| Exon 8 | c.994C>T | p.Arg332X | Truncating mutation |
| Exon 8 | c.1001T>G | p.Leu334X | Truncating mutation |
| Exon 8 | c.1099_1103del | p.Glu368ArgfsX2 | Truncating mutation * |
| Exon 9 | c.1274dup | p.Gly426ArgfsX22 | Truncating mutation |
| Exon 10 | c.1348C>T | p.Gln450X | Truncating mutation |
| Exon 10 | c.1366delinsCA | p.Glu456GlnfsX15 | Truncating mutation * |
| Exon 10 | c.1392delA | p.Ala465ProfsX9 | Truncating mutation |
| Exon 10 | c.1392delA | p.Ala465ProfsX9 | Truncating mutation |
| Exon 10 | c.1401del | p.Glu468LysfsX6 | Truncating mutation |
| Exon 11 | c.1424C>A | p.Ser475X | Truncating mutation |
| Exon 12 | c.1771C>T | p.Arg591X | Truncating mutation |
| Exon 12 | c.2521_2522dupCA | p.Arg842IlefsX18 | Truncating mutation |
| Exon 12 | c.2617C>T | p.Arg873X | Truncating mutation |
| Exon 12 | c.2617C>T | p.Arg873X | Truncating mutation |
| Exon 12 | c.2617C>T | p.Arg873X | Truncating mutation |
| Exon 12 | c.2617C>T | p.Arg873X | Truncating mutation |
| Exon 12 | c.2617C>T | p.Arg873X | Truncating mutation |
| Exon 12 | c.2617C>T | p.Arg873X | Truncating mutation |
| Exon 12 | c.2695C>T | p.Arg899X | Truncating mutation |
| Intron 2 | c.248-1G>A | Splice defect | Splice defect |
| Intron 3 | c.418+3A>T | Splice defect | Splice defect |
| Intron 3 | c.418+3A>T | Splice defect | Splice defect |
| Intron 3 | c.418+3A>T | Splice defect | Splice defect |
| Intron 6 | c.852+1G>A | Splice defect | Splice defect |
| Intron 6 | c.853-1G>C | Splice defect | Splice defect |
| Intron 6 | c.853-2A>G | Splice defect | Splice defect |
| Intron 7 | c.967+5G>T | Splice defect | Splice defect |
| Intron 9 | c.1276+3A>T | Splice defect | Splice defect |
| Intron 9 | c.1277-9A>G | Splice defect | Splice defect |
| Intron 9 | c.1277-9A>G | Splice defect | Splice defect |
| Intron 10 | c.1413+1G>A | Splice defect | Splice defect |
| Deletion of exon 1 to 4 | Deletion of exon 1 to 4 | Deletion of exon 1 to 4 | Large rearrangement |
| Deletion of exon 1 to 13 | Deletion of exon 1 to 13 | Deletion of exon 1 to 13 | Large rearrangement |
| Deletion of exon 1 to 3 | Deletion of exon 1 to 3 | Deletion of exon 1 to 3 | Large rearrangement |
| Deletion of exon 2 | Deletion of exon 2 | Deletion of exon 2 | Large rearrangement |
| Duplication of exon 4 to 8 | Duplication of exon 4 to 8 | Duplication of exon 4 to 8 | Large rearrangement |
| Deletion of exon 6 | Deletion of exon 6 | Deletion of exon 6 | Large rearrangement |
| Deletion of exon 8 | Deletion of exon 8 | Deletion of exon 8 | Large rearrangement |
| Deletion of exon 8 | Deletion of exon 8 | Deletion of exon 8 | Large rearrangement |
| Deletion of exon 8 | Deletion of exon 8 | Deletion of exon 8 | Large rearrangement |
| Deletion of exon 10 | Deletion of exon 10 | Deletion of exon 10 | Large rearrangement |
| Deletion of exon 10 | Deletion of exon 10 | Deletion of exon 10 | Large rearrangement |
| Deletion of exon 10 | Deletion of exon 10 | Deletion of exon 10 | Large rearrangement |
| Deletion of exon 10 | Deletion of exon 10 | Deletion of exon 10 | Large rearrangement |
| Deletion of exon 10 | Deletion of exon 10 | Deletion of exon 10 | Large rearrangement |
| Deletion of exon 10 | Deletion of exon 10 | Deletion of exon 10 | Large rearrangement |
| Deletion of exon 10 | Deletion of exon 10 | Deletion of exon 10 | Large rearrangement |
| Deletion of exon 11 to 12 | Deletion of exon 11 to 12 | Deletion of exon 11 to 12 | Large rearrangement |
| Deletion of exon 11 to 13 | Deletion of exon 11 to 13 | Deletion of exon 11 to 13 | Large rearrangement |
| Deletion of exon 11 to 13 | Deletion of exon 11 to 13 | Deletion of exon 11 to 13 | Large rearrangement |
| Deletion of exon 11 to 13 | Deletion of exon 11 to 13 | Deletion of exon 11 to 13 | Large rearrangement |

* Truncating mutation predicted to escape NMD
